# Methylome Patterns of Cattle Adaptation to Heat Stress

**DOI:** 10.3389/fgene.2021.633132

**Published:** 2021-05-28

**Authors:** Marcello Del Corvo, Barbara Lazzari, Emanuele Capra, Ludmilla Zavarez, Marco Milanesi, Yuri Tani Utsunomiya, Adam Taiti Harth Utsunomiya, Alessandra Stella, Guilherme de Paula Nogueira, Josè Fernando Garcia, Paolo Ajmone-Marsan

**Affiliations:** ^1^Department of Animal Science Food and Nutrition – DIANA, Nutrigenomics and Proteomics Research Centre – PRONUTRIGEN, and Biodiversity and Ancient DNA Research Centre, Università Cattolica del Sacro Cuore, Piacenza, Italy; ^2^Istituto di Biologia e Biotecnologia Agraria, Consiglio Nazionale delle Ricerche IBBA CNR, Milan, Italy; ^3^School of Veterinary Medicine, Araçatuba, Department of Production and Animal Health, São Paulo State University (unesp), Araçatuba, Brazil; ^4^International Atomic Energy Agency, Collaborating Centre on Animal Genomics and Bioinformatics, Araçatuba, Brazil

**Keywords:** heat stress, epigenetics, DNA methylation, animals welfare, cattle, red blood cell

## Abstract

Heat stress has a detrimental impact on cattle health, welfare and productivity by affecting gene expression, metabolism and immune response, but little is known on the epigenetic mechanisms mediating the effect of temperature at the cellular and organism level. In this study, we investigated genome-wide DNA methylation in blood samples collected from 5 bulls of the heat stress resilient Nellore breed and 5 bulls of the Angus that are more heat stress susceptible, exposed to the sun and high temperature-high humidity during the summer season of the Brazilian South-East region. The methylomes were analyzed during and after the exposure by Reduced Representation Bisulfite Sequencing, which provided genome-wide single-base resolution methylation profiles. Significant methylation changes between stressful and recovery periods were observed in 819 genes. Among these, 351 were only seen in Angus, 366 were specific to Nellore, and 102 showed significant changes in methylation patterns in both breeds. KEGG and Gene Ontology (GO) enrichment analyses showed that responses were breed-specific. Interestingly, in Nellore significant genes and pathways were mainly involved in stress responses and cellular defense and were under methylated during heat stress, whereas in Angus the response was less focused. These preliminary results suggest that heat challenge induces changes in methylation patterns in specific loci, which should be further scrutinized to assess their role in heat tolerance.

## Introduction

Rapid and unpredictable climate change and extreme climatic events (floods, drought, extreme temperatures) are increasing in frequency, which have an impact on agricultural productivity and affect food security. These changes are not only limited to tropical and subtropical regions but also affect more temperate regions (Rajaud and Noblet-Ducoudré, 2017). In the most vulnerable areas, management and breeding of livestock should address heat stress and decreasing water availability, while maintaining fitness and productivity in order to remain viable (Rojas-Downing et al., 2017). To satisfy the demand for animal products, which is increasing at a faster rate than human population growth, it is necessary for the livestock sector to respond to rapid changes in the climate.

Heat stressed animals are not able to adequately dissipate the excess of endogenous and exogenous heat to maintain the body thermal balance (Bernabucci et al., 2014). Heat stress is a primary stressor for high-producing dairy cows which have a high metabolic rate with associated endothermy, which results in heat-induced depression of milk production and growth (Cowley et al., 2015). Therefore, heat stress results in economic losses due to reduced production and reproduction performance (Aguilar et al., 2010; Nardone et al., 2010; Mehla et al., 2014; Biffani et al., 2016; Macciotta et al., 2017). Physiological changes are observed affecting the ability of the animal to cope with heat stress. For example, rectal temperature and respiration rate increase (Dikmen et al., 2015; Perano et al., 2015). Some of these reponses have a genetic component, moderate heritability and have been associated with genomic variants, e.g., several candidate genes have been identified for rectal temperature (Dikmen et al., 2013, 2015). Traditional phenotype-based selection for adaptation and fitness, while maintaining productivity, is difficult and slow as these traits are difficult to measure. For this reason, genomic information could accelerate the genetic progress for adaptation traits. SNP markers associated with heat tolerance have been detected in dairy cattle (Hayes et al., 2009; Dikmen et al., 2012, 2013), although few causative genes have been identified (reviewed in Collier et al., 2008). One of these, the prolactin receptor gene (*PRLR*), carries mutations having a major effect on heat tolerance (Littlejohn et al., 2014; Porto-Neto et al., 2018). This variant causes the SLICK phenotype which is easily detected as it confers animals a short and sleek coat (Olson et al., 2003; Dikmen et al., 2008). Holstein cattle into which a SLICK haplotype was introduced had superior thermoregulatory ability compared with wild-type Holsteins (Dikmen et al., 2014).

Little is known about the biology of adaptation, and even less about mechanisms that mediate changes in gene expression and metabolism in animals subjected to environmental stress. Technologies developed to sequence and characterize the human genome have paved the way for the routine sequencing of the genomes of agriculturally important species. In addition to the genome sequence, epigenetic features control gene expression and have attracted much attention in the last few years. At least four molecular systems are involved in the control of gene expression, including DNA methylation (Bernstein et al., 2007), non-coding RNAs (Mitchell et al., 2009), histone post-translational modifications (Strahl and Allis, 2000), and chromatin remodeling (Cosma et al., 2010). DNA methylation was the first identified and is currently the better-studied epigenetic regulatory mechanism of gene expression. In mammalian cells, DNA methylation mostly occurs at the cytosine of a CpG dinucleotide, and CpG islands, which are enriched for CpG dinucleotides, are frequently located in promoter regions at the 5′ of coding sequences.

There is still limited knowledge on the relationship between epigenetic patterns and phenotypic variation in livestock. Nevertheless, epigenetic effects on some trait have been described, e.g., growth is affected by epigenetic imprinting of IGF2 in pig (Van Laere et al., 2003), and epigenetic regulation of callipyge affects development in sheep (Georges et al., 2003; Vuocolo et al., 2007). Recent studies have characterized pig and chicken methylomes (Li et al., 2012; Nätt et al., 2012). An atlas of the porcine methylome identified that differentially methylated regions contain ∼80% of the known or candidate human obesity-related genes, 72% of which mapping in QTL regions that affect fatness and pork quality (Li et al., 2012). Nätt et al. (2012) suggested that the heritability of methylation in the chicken genome might play a role in adaptation and selection since they observed an over-representation of differentially methylated genes in selective sweep regions associated with domestication. This could mean that novel methylation patterns have been acquired by domestic animals during their selection history. In dairy cows, recent work has shed light on the role of epigenetics in the regulation of milk synthesis and mammary development (Singh et al., 2010). In the lactating cow, remethylation of the typically hypo-methylated casein gene promoter drastically reduces casein mRNA expression and milk protein synthesis during acute udder infection.

The association between DNA methylation patterns and heat stress has been suggested. For example exposure of male guinea pigs to chronic heat stress has been shown to alter DNA methylation patterns in the liver of both the F0 and F1 generations (Weyrich et al., 2016).

In the current paper, epigenomic responses to heat stress were investigated in indicine (*Bos indicus*) and taurine (*Bos taurus*) cattle. We hypothesize that, in addition to morphological traits determined by genetics (coat color, body size and conformation, large ears, loose skin, sweat gland size), the adaptation to heat and the better ability to cope with deleterious effects of a high temperature of the indicine Nellore compared to the taurine Angus breed is also associated with changes in DNA methylation patterns of specific regulatory genes and metabolic pathways. In the study reported here, cattle of these two breeds were exposed to the sun during the high temperature and high humidity season of the Brazilian South-East region and the physiological and epigenetic responses assessed.

## Materials and Methods

### Animals Treatment and Experimental Design

**FIGURE 1:**
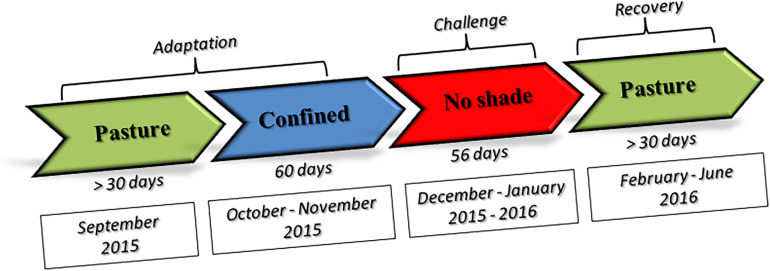
Schematic representation of the experimental design.

A total of 25 Nellore (indicine heat-tolerant breed) and 25 Angus (taurine heat-susceptible breed) bullocks were included in the investigation. Animals were all healthy young males of about 15 months of age at the time of the investigation. We selected half-sibs animals within each breed to minimize genetic variation. Angus bullocks were purchased at 7 months of age in Uruguaiana (Rio Grande do Sul state, Brazil) and arrived at UNESP Aracatuba (Sao Paulo state, Brazil) experimental station (located at −21.186244 latitude and −50.439053 longitude) on June 13, 2015. Seven-month old Nellore bullocks were purchased in Dourados (Mato Grosso do Sul state, Brazil), kept at Agua Branca farm in Birigui (Sao Paulo, Brazil) until 10 months of age and moved to UNESP on September 2, 2015. Animals were kept at the experimental station of the Veterinary Faculty, Universidade Estadual Paulista – UNESP- Araçatuba Campus. A full description of the diet fed during the study is given in Supplementary File 1, while a schematic representation of experimental design is given in the Figure 1.

***Adaptation period***. On October 3, 2015, the steers were randomly assigned to 2 groups (1 Nellore and 1 Angus groups) of 12 or 13 animals and kept in two 200 square meter paddocks of which 100 square meters was covered by a shadowing net (80% sunblock) with regular access to pasture until December 3 (60 days). This period will be further referred to as pre-challenge adaptation period (PRE).

***Challenge.*** The experiment started on December 4, 2015, by removing the shadowing net from paddocks. Animal groups were therefore kept without shade until February 3, 2016 (56 days). This period is subsequently referred to as the experimental challenge period (EXP).

***Recovery period.*** On February 4th shadowing nets were replaced such that all animals were kept with shade available and were allowed access to pasture until slaughter at the end of June 2016. This period will be further referred to as post challenge recovery period (POST).

***Sampling.*** Nellore adaptation period terminated in November 2015, when the season was already hot and humid and animals were under moderate stres. Sampling was therefore planned at the beginning of February 2016, at peak heat stress period, and in the middle of June 2016, during the cool season, after full recovery from heat stress.

### Environmental Data Collection

Environmental data were collected hourly from June 1, 2015 to October 10, 2016 from CETESB meteorological station located in the UNESP campus, nearby the experimental site. Experimental variables collected were Temperature (T, in centigrades) and Humidity (H, in percentage). Missing data were imputed using the median among −10/ + 10 day data at the same time.

### DNA Isolation, Library Preparation and Sequencing

Blood (10 ml) was collected from the same 5 randomly selected Nellore (heat stress resilient) and 5 randomly selected Angus (heat stress susceptible) belonging to sun-exposed group, in two periods: in February 2016 (stressful/challenge period) and June 2016 (recovery period). We used QIAamp DNA Blood Midi Kit (Qiagen) procedures to extract DNA from whole blood. One μg input was used in the MSP1 digest by overnight incubation at 37°C, following manufacturer instruction. Libraries were prepared using the TruSeq^®^ DNA PCR-Free Library Preparation Kit (Illumina) including a step of bisulfite treatment. Subsequently, ligated products corresponding to DNA fragments 150–400-bp long were converted with EpiTectBisulfite Kits (Qiagen) and finally, PCR amplified with KAPA HiFi Uracil + (KapaBiosystems) to obtain RRBS libraries. DNA with a known methylation level was used as a spike control, and all conversion rates were >99%, ranging from 99.11 to 99.53% (Supplementary Table 1). Twenty Reduced Representation Bisulfite Sequencing (RRBS) libraries were used for cluster generation and subsequent sequencing on Illumina HiSeq 2500 PE 2 × 50 bp (NXT-Dx Ghent, Belgium).^1^

### Environmental Indexes

From environmental data we obtained THI (*Temperature Humidity Index*), which considers temperature (T) and humidity (H) in a single index: (1.8 ^∗^ T + 32) − [(0.55 − 0.0055 ^∗^ H) ^∗^ (1.8 ^∗^ T - 26)] (Thom, 1959). Values above 75 were considered as moderate stress, above 85 as severe stress and over 90 as extreme stress (McDowell et al., 1976).

### Data Analysis

Preliminary quality control of raw reads was carried out with FastQC v0.11.9.^2^ The FASTQ sequence reads were generated using the Illumina Casava pipeline 1.8.2. The quality and adapter trimming of Illumina raw sequenze was performed with Trim Galore v0.6.1^3^ using a two-step approach, which allowed us to remove two additional bases containing a cytosine, which were artificially introduced in the end-repair step during the library preparation. Bismark software (version 0.22.3) (Krueger and Andrews, 2011) was used to align each bisulfite-treated read to the bovine reference genome (ARS-UCD1.2) with option-N 1 (maximum number of mismatches allowed). The reference genome was first transformed into a bisulfite-converted version (C-to-T and G-to-A conversions) and then indexed using bowtie2 software (Langmead and Salzberg, 2012). Sequence reads were also transformed into fully bisulfite-converted versions (C-to-T and G-to-A conversions) before they were aligned to similarly converted versions of the genome in a directional manner. Sequence reads that produced the best unique alignment from the two alignment processes (original top and bottom strand) were then compared to the normal genomic sequence, and the methylation state of all cytosine positions in the reads was inferred using the *Bismark_methylation_extractor* function. Read duplicates were marked and removed using Picard Tools v2.23.^4^

### Identification of Differentially Methylated Regions (DMRs) and Genes (DMGs)

DMRs were identified within the statistical environment R using the library methylKit (Akalin et al., 2012), which applies a sliding-window approach with a window of 1,000 bp and a step size of 500 bp. We first filtered out bases with less than 10 reads or more than the 99.9th percentile of coverage distribution. This step was done to ensure a good quality of the data and great confidence in the methylation percentage. Coverage values were then normalized by default and bases were merged in order to retain the ones that were covered in all samples. A logistic regression was then implemented to obtain *p* values. Specifically, in any given region methylKit models the methylation proportion P_*i*_, for sample *i* = 1,…,*n* (where n is the number of animals) by the following logistic regression model:

$$\log\left(P_{i}/\left(1-P_{i}\right)\right)=\beta_{0}+\beta_{1}*T_{i}$$

where T_*i*_ denotes the treatment indicator for sample i, T_*i*_ = 1 if sample i is in the treatment group (stress period) and T_*i*_ = 0 if sample i is in control group (recovery period). The parameter β_0_ denotes the log odds of the control group and β_1_ the log oddsratio between the treatment and control group. Therefore, independent tests for all the regions of interest are run against the null hypothesis H_0_: β_1_ = 0. The null hypothesis is rejected when the logodds (and hence the methylation proportions) are different between the treatment and the control group and the region therefore classified as a DMR. *P* values were then adjusted to *q* values using the Sliding Linear Model (SLIM) method (Wang et al., 2011). We excluded covariates and over dispersion correction from the model since they showed not to provide significant changes to the results. DMRs were considered statistically significant if the *q* value was lower than 0.05, read coverage greater than 10 in all samples and methylation difference between groups at least 15%. When a DMR and a specific gene region overlapped, the corresponding gene was selected as the DMR-related gene, namely a differentially methylated gene (DMG). Gene features within DMGs were annotated by matching information available in the General Feature Format (GFF) file downloaded from the Ensembl database, release 101.^5^ The promoter region was defined as an area 2-kb upstream of the transcription start sites.

### Gene Ontology and KEGG Enrichment Analysis of DMR-Related Genes

Gene Ontology (GO) and KEGG pathway enrichment analysis of DMGs were performed by *clusterProfiler*, an ontology-based R package able to automate the process of biological term classification and enrichment analysis of gene clusters and to provide a visualization module for displaying analysis results (Yu et al., 2012). GO terms and KEGG with *p* values of less than 0.01 and *q* values of less than 0.1 were considered significantly enriched by DMR-related genes.

## Results

### Heat Stress Based on Environmental Parameters

**FIGURE 2:**
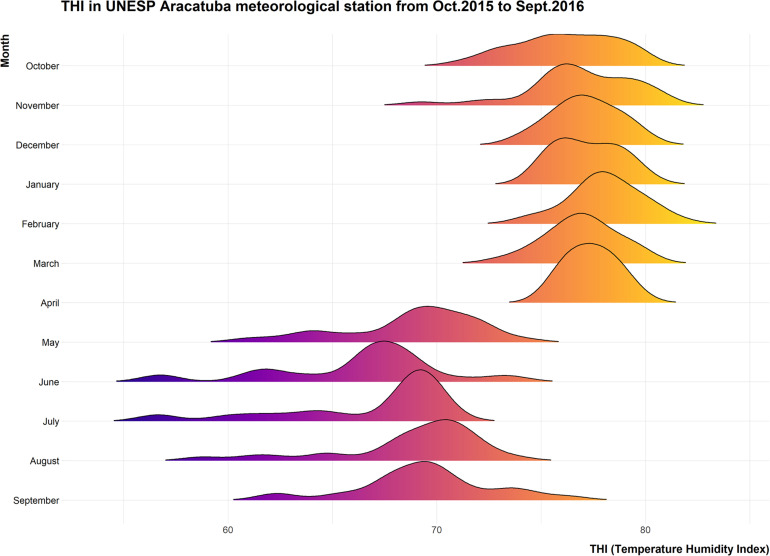
Ridgeline plot showing distribution of daily average THI recorded at CETESB meteorological station located in the UNESP campus from October 2015 to September 2016.

The environmental conditions were analyzed and Temperature Humidity indices (THI), were calculated to evaluate the severity of heat challenge before during and after the experimental period (Figure 2).

### Global Mapping of DNA Methylation

A mean of 60 million raw reads were generated for Nellore samples and 67 million for Angus samples during challenge period and 89 million and 80 million, respectively during the recovery period. After removing low-quality reads, 57 and 64 million of clean reads for the experimental period and 85 and 76 million for the recovery period for Nellore and Angus respectively remained. In the two periods the average value of uniquely mapped reads covering the cattle genome corresponds to 43.18 and 43.82% in Nellore and 44.5 and 46.1% in Angus (Table 1 and Supplementary Table 1).

**TABLE 1:** Data generated by Reduced Representation Bisulfite Sequencing (RRBS).

	Nellore		Angus	
	Challenge	Recovery	Challenge	Recovery
Raw reads	60,091,512	89,179,323	67,901,537	80,179,472
Clean reads	57,699,800	85,587,160	64,763,022	76,551,213
Total mapped reads	25,980,704	38,923,431	30,123,254	36,899,326
Average mapping rate (%)	43.18%	43.82%	44.5%	46.1%

### DNA Methylation Patterns

We observed an average genome-wide level of 66.26% CG, 0.44% CHG, and 0.32% CHH methylation (H = A, T or C) in Nellore during challenge and 66.8% CG, 0.5% CHG, and 0.34% CHH methylation during recovery. Values in Angus were 67.7% CG, 0.3% CHG, and 0.2% CHH methylation in the experimental period and 67.14% CG, 0.3% CHG, and 0.22% CHH methylation in the post challenge period (Table 2).

**TABLE 2:** Genome-wide percentage of methylated CG, CHG, and CHH in Nellore and Angus breed during challenge and recovery period.

	Nellore		Angus	
	Challenge	Recovery	Challenge	Recovery
mCG percent (%)	66.26%	66.8%	67.7%	67.14%
mCHG percent (%)	0.44%	0.5%	0.3%	0.3%
mCHH percent (%)	0.32%	0.34%	0.2%	0.22%

Over 66% of the CpG sites were methylated in both breeds during challenge and recovery periods. Heat stress effects did not significantly change DNA methylation of either the CpG and non-CpG sites in both breeds at a genome-wide level (Table 2).

### DNA Methylation Level in Different Genome Features

We investigated the DNA methylation level in five different genome features including promoters, 5′UTRs, exons, introns and 3′UTRs, using a sliding window approach. In both breeds methylation level of CG dinucleotides showed a similar pattern during challenge and recovery periods. Promoter regions exhibited a higher variability compared with other features, with the maximum distance between the first and the third quartile. Exon, intron and 3′UTR regions showed the highest methylation level, while the 5′UTR had the lowest average level of methylation (Supplementary Figure 1).

### Differentially Methylated Regions (DMRs) and Related Genes (DMGs)

**FIGURE 3:**
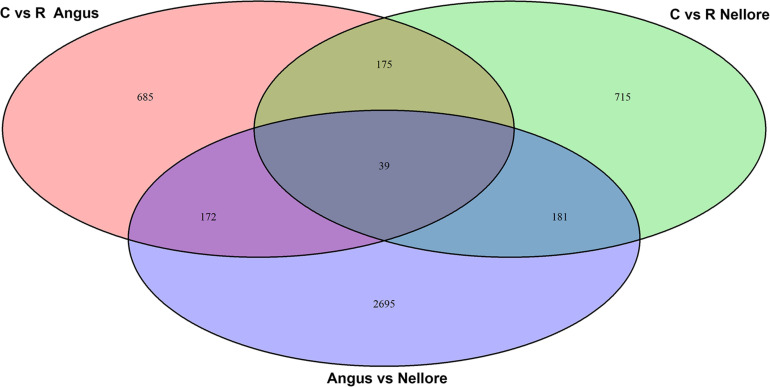
Venn diagram of differentially methylated windows in Angus and Nellore (C, Challenge, R, Recovery).

Principal component analysis (PCA) based on genome-wide DNA methylation clearly distinguished Angus from Nellore but not the two different periods (stress and recovery) (Supplementary Figure 2). Differential methylation analysis identified a total of 4662 DMRs. There were 2695 DMRs between Angus and Nellore that did not change between stress and recovery periods and therefore are related to breed differences rather than a response to heat. It is interesting to note that 62% of these were hyper-methylated and 38% hypo-methylated in Angus compared with Nellore, suggesting an overall higher level of metylation in Angus vs Nellore. A total of 1967 windows were differentially methylated in the stress period compared with the recovery period (Supplementary Table 2), 857 in Angus, 896 in Nellore of these only 214 were seen in both breeds, indicating that epigenetic signatures related to heat stress response and recovery are mostly breed specific (Figure 3).

**FIGURE 4:**
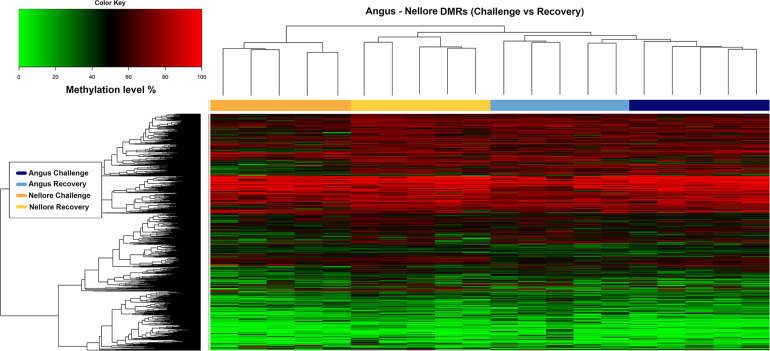
Heatmap of methylation level (y axis) in Angus and Nellore populations (x axis) within 1967 DMRs. The blue and azure bars correspond to methylation level of Angus samples during challenge and recovery season and the orange and yellow bars correspond to methylation level of Nellore samples during challenge and recovery season respectively.

Heat map analysis indicates that in Angus, a large proportion of methylated regions were maintained in both periods possibly indicating difficulty in recovering after a severe stress. Conversely, Nellore seems to respond to stress with a demethylation of several key genomic regions during challenge (Figure 4).

The blue and azure bars correspond to methylation level of Angus samples during challenge and recovery season and the orange and yellow bars correspond to methylation level of Nellore samples during challenge and recovery season respectively.

We annotated all 1967 DMRs using the genomic location and the matching annotation information on the genome structure of the cattle genome. The 857 DMRs containing windows identified in Angus overlapped with 351 annotated genes (192 hyper-methylated and 159 hypo-methylated during stress), while in Nellore the 896 windows overlapped with 366 genes (85 hyper-methylated and 281 hypo-methylated during stress). Among these genes 39 were common between Angus and Nellore even though they mapped to different genomic regions. Furthermore the 214 DMRs that were shared between breeds overlapped with 102 genes (among these, 61% were hyper or hypo-methylated in both breeds, while 39% show an opposite trend). In both breeds more than 50% of the differentially methylated regions were located in introns, 31% in the exons, withless than 5% in each of 3′,5′ UTR and promoter regions.

### GO and KEGG Analysis of DMGs

**FIGURE 5:**
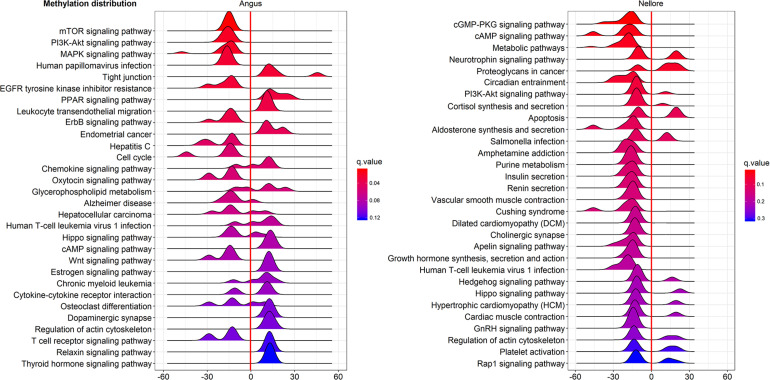
KEGG analysis of DMGs in Angus (left side) and Nellore (right side) in challenge and recovery periods. Peak hight is proportial to the number of differentially methylated genes, color to *p* value. Negative values indicate hypo-methylation, positive values hyper-methylation during heat stress.

**FIGURE 6:**
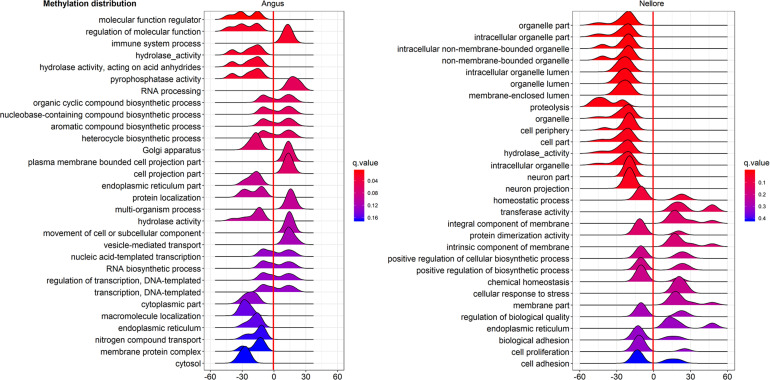
Gene Ontology analysis of DMGs in Angus (left side) and Nellore (right side) in challenge and recovery periods. Peak hight is proportial to the number of differentially methylated genes, color to *p* value. Negative values indicate hypo-methylation, positive values hyper-methylation during heat stress.

Pathway enriched analysis was carried out to identify the metabolic and the immune system pathways affected and identify those that are relevant to a heat stress response. For each breed all DMGs detected were mapped to terms within the GO and the KEGG databases. Both breeds had a similar proportion of DMGs within GO pathways enriched: cellular process (Angus 184; 61.5%; Nellore 188; 62.7%); single-organism process (Angus 73; 24.4%; Nellore 68; 22.7%) and metabolic process (Angus 42; 14%; Nellore 44; 14.7%). KEGG analysis identified 77 pathways in Angus and 78 in Nellore. Among theme, 49 overlap between the two breeds, but a comparison between the top 30 pathways (Figures 5, 6) showed only few cellular functions (a single one in GO enrichment analysis, phosphoric diester hydrolase activity) in common between breeds. It is also interesting to note that shared pathways are enriched only minimally by the same DMGs (32% in Angus and 30% in Nellore), and more than half of these presents opposite behavior, having higher methylation in the stress period and lower in the recovery period in one breed and the opposite in the other. This reflects again the specific response that we previously observed from the Venn diagram analysis.

## Discussion

The tropical environment of the Brazilian Southeast region is characterized by high temperature and humidity and episodes of feed and water scarcity. Nellore cattle have greater resistance to such conditions, surviving, breeding and being productive in the tropics (Valente et al., 2015). In comparison Angus have difficulties adapting to extreme climatic conditions, failing to thrive with tropical heat and humidity, and are susceptible to pathogen challenges. Animal homeostasis in different environments is maintained by modulating metabolic processes to counteract the deleterious effects of stressful conditions (Chovatiya and Medzhitov, 2014). Epigenetic signatures, such as DNA methylation, act as a mediator between changing environmental conditions and animal metabolic response to maintain homeostasis. The present work investigated: (i) changes in DNA methylation that occur in cattle under heat stress, compared with non-stressful conditions and; (ii) differences in methylation in response to heat stress observed in tolerant indicine Nellore versus non-tolerant taurine Angus cattle.

We analyzed genome-wide DNA methylation using the RRBS method. We extracted DNA from blood, as an easily accessible and highly informative indicator of animal response to environmental challenges. The main source of DNA was therefore obtained from white cells, even if we can’t exclude a possible co-extraction of some circulating free DNA, that under stressful conditions may increase its usually low concentration in plasma (Cree et al., 2017; Henriksen et al., 2020). It has been shown that methylation levels at CpG sites in leukocytes are predictors of variability of CpG methylation or may at least partially be considered as proxies of epigenetic processes in other tissues (Ma et al., 2014; Braun et al., 2019; Del Corvo et al., 2020).

### Key Pathways Related to the Immune System and Metabolic Processes in Angus and Nellore

In the present study several KEGG gene-sets with differential methyation were identified both in Nellore and Angus breeds in response to heat stress. In Nellore we detected metabolic pathways, circadian entrainment, cGMP-PKG, cAMP and PI3K-Akt signaling pathways that were enriched in differentially methylated genes. Several of these pathways are related to the immune system and inflammatory response. Circadian entrainment pathway has emerged as an internal regulator with the potential to impact disease (Scheiermann et al., 2013). Furthermore, a previous study demonstrated that signaling pathways (cGMP-PKG, cAMP and PI3K-Akt) might form a network with genes related to immuno regulatory mechanisms (Zaccolo and Movsesian, 2007; Wehbi and Taskén, 2016).

In the current study we observed gene enrichment in KEGG metabolic pathways in blood that are known to be involved in heat stress response in other tissues (Rhoads et al., 2010). In particular, pathways for hormone synthesis and/or secretion (cortisol, aldosterone, insulin, renin, growth hormone, GnRH; Figure 6) were all enriched in hypo-methylated genes. Blood cells have been previosly shown to be indicative of methylation status of other tissues in human (Ma et al., 2014). This characteristic is to be further confirmed, however, it would be remarkable, as blood is an easily accessible tissue.

In Angus patterns of response were detected from pathway analysis, that would not have been detectable in a single gene analysis. Several KEGG pathways were enriched in DMGs during heat exposure, including signaling networks, such as MAPK, mTOR, ErbB, PI3k/AKT and pathways related to hepatitis C and other viral infections that can be stimulated in response to heat stress (Danaher et al., 1999). A study of rat skeletal muscle demonstrated that genes in mTOR and PI3k/AKT pathways are activated by a temperature increase (Yoshihara et al., 2013). The ErbB signaling pathway plays a role upstream of MAPK signaling, which affects lipid metabolism (Zhang et al., 2017). Heat stress affects the regulation of lipolysis and the rate-limiting enzymes of lipogenesis (Faylon et al., 2015). In addition, the MAPK pathway is a downstream target of ErbB receptors, which are mediators of cell proliferation, differentiation, apoptosis, and cell motility (Holbro and Hynes, 2004).

Furthermore phosphoric diester hydrolase activity, the only GO pathway shared by both breeds as shown by enrichment analysis, seems to be activated by differentially methylated genes in liver and mammary gland of bull calves in another study as a consequence of maternal conditions of heat stress (Skibiel et al., 2018).

### Key Differentially Methylated Genes Related to the Immune System and Inflammatory Response in Angus and Nellore

We identified several genes involved in the biological processes significant for the immune system and inflammatory response. Among them fatty acid elongase 5 (*ELOVL5)*, F2R like thrombin or trypsin receptor 3 (*F2RL3)*, fatty acid desaturase 1 (*FADS1)*, mitogen-activated protein kinase kinasekinase 1 (*MAP3K1)*, phosphodiesterase 5A (*PDE5A)*, netrin 1 (*NTN1)*, RAS p21 protein activator 3 (*RASA3)* and peroxiredoxin 1 (*PRDX)* were identified in Nellore. Genes linked to immune defense were in DMRs between stress and recovery periods also in Angus. These included autophagy related 16 like 2 (*ATG16L2)*, calcium voltage-gated channel subunit alpha1 C (*CACNA1C)*and growth arrest and DNA damage inducible alpha (*GADD45A)*. These genes are expressed in blood cells and have been shown to be differentially methylated also in the brain and are linked to nervous system functions.

In the present study, DMGs identified in Nellore were mainly hypo-methylated in the heat stress period, including in prometer regions, suggesting a pro-active response of this breed to the challenge, to maintain homeostasis.

For example, F2R like thrombin or trypsin receptor 3 (*F2RL3)* encodes the thrombin protease-activated receptor-4 *(PAR-4)*, which is expressed on the surface of various body tissues, including circulating leukocytes (Xu et al., 1998; Vergnolle et al., 2002). The activation of PAR-4 is involved in leukocyte recruitment, as well as in the regulation of vascular endothelial cell activity (Vergnolle et al., 2002; Kataoka et al., 2003; Leger et al., 2006; Gomides et al., 2012). These pathophysiological events are considered to be the early steps of the vascular inflammatory reaction (Vergnolle et al., 2002; Steinhoff et al., 2005; Leger et al., 2006). Sibbons et al. (2018) showed that in peripheral blood mononuclear cells (PBMCs), the up-regulation of enzymes coded by the fatty acid elongase 5 *(ELOVL5)* and fatty acid desaturase 1 (*FADS1)* genes increases the synthesis of n-3 PUFA. Polyunsaturated fatty acids (PUFAs) play key roles in the immune response by acting as substrates for the synthesis of lipid second messengers, including eicosanoids, implicated in cell activation, and for cell membrane biosynthesis. In the present study, these two genes were hypo-methylated during the challenge period, possibly indicating an increase in gene expression. Interestingly, supplementation of PUFAs to the diet can protect animals from the deleterious effects of heat stress on bull sperms by improving quality and motility parameters of fresh semen (Gholami et al., 2011).

A mitogen-activated protein kinase (*MAP3K-1*) which is member of family of serine/threonine kinases (Pham et al., 2013) was in a region that was hypo-methylated in Nellore during the heat stress period. MAPK signaling is important for T lymphocyte development, homeostasis, and effector responses (von Boehmer, 1990; Kronenberg and Gapin, 2002). Among these, natural killer T cells (NKT) constitute a unique T cell population of the immune system (Kronenberg and Gapin, 2007). In mice, it was demonstrated that a deletion of MAP3K-1 leads to an increased NKT cell infiltration into the liver and a higher degree of liver damage (Suddason et al., 2016). It is therefore not surprising that in Nellore this gene is involved in heat response, since it has been widely documented in cattle that acute thermal stress decreases the number of helper T cells and increases the number of NK cells (Nagai and Iriki, 2001).

Phosphodiesterase 5A (*PDE5A)* encodes a cGMP-specific phosphodiesterase involved in cell signaling, protein binding, phosphorylation, cell activation and cGMP binding was found to be hypo-methylated in Nellore during heat stress. A hypo-methylated window located within an intronic region of this gene has been described in the above mentioned work of Skibiel et al. (2018), where DNA methylation of bull calves and heifers has been measured after gestation under maternal conditions of heat stress. In the present study another intronic region of the same gene was also hypo-methylated.

Both RAS p21 protein activator 3 (*RASA3)* and netrin 1 (*NTN1)* were hypo-methylated in Nellore during heat stress. RASA3 encodes for an enzyme member of the GAP1 family of GTPase-activating proteins. This gene is highly expressed in peripheral blood mononuclear cells during activation of the immune system cells and the inflammatory reaction (Wu et al., 2018). NTN1 is a neuroimmune guidance protein expressed in vascular endothelial cells that regulates inflammatory cell recruitment (Ly et al., 2005; Boneschansker et al., 2016). In human, a significant increase in core body temperature during heat stress is associated with a worsening of cognitive functions of people coping with mental illness (Hämäläinen et al., 2012). Differential methylation of both NTN1 and RASA3 has been reported in blood mononuclear cells of patients affected by the neurodegenerative disease, multiple sclerosis (MS), a disease known to be influenced by high body temperature (Flensner et al., 2011). This suggests that epigenetic regulation of NTN1 and RASA3 may be involved in homoeostatic changes of specific nervous system functions due to heat stress. Multiple sclerosis is associated with systemic sclerosis (SSc) (Pelidou et al., 2007). A recent methylome and transcriptome study carried out in blood cells of patients affected by systemic sclerosis detected five DMGs that were hypo-methylated in heat challenged Nellore in the present study: inositol polyphosphate-5-phosphatase A (*INPP5A)*, Spi-1 proto-oncogene (*SPI1)*, transcription factor 3 (*TCF3)*, in addition to F2RL3 and MAP3K1 (Zhu et al., 2018). This suggest a link between the increase of body temperature and changes in the nervous system, through regulation of inflammatory processes.

Heat stress induced oxidative stress (Altan et al., 2003; Mujahid et al., 2005; Lin et al., 2006) can damage cellular macromolecules and interfere with cell signaling pathways. Peroxiredoxin1 (*PRDX1*) encodes a member of the peroxiredoxin family, which acts as a peroxidase as well as a chaperone to protect proteins from oxidative damage (Nassour et al., 2016) and plays key roles in innate immunity and inflammation (Knoops et al., 2016). The effect of heat stress on *in vitro* bovine granulosa cells resulted in incresed expression of PRDX1 in response to oxidative stress and to re-establish cellular homeostasis (Alemu et al., 2018). PRDX1 was hypo-methylation within intronic regions during the challenge period in Nellore.

The level of methylation of Angus samples was higher than those of Nellore, more than half of the significant DMGs were hyper-methylated in the promoter regions during heat stress and therefore gene expression of these genes was likely to be reduced in response to the challenge. This may be associated with the greater vulnerability of this breed to heat stress compared to the Nellore (Bagath et al., 2019). Some of the DMGs were associated with the immune system and were hyper-methylated during heat stress. Autophagy related 16 like 2 (*ATG16L2)* showed opposite behavior in the two breeds, having higher methylation in the stress period and lower in the recovery period in Angus and the opposite in Nellore. ATG16L2 codes for a protein which plays a role in autophagy (Ishibashi et al., 2011). It was observed that heat stress in pig populations leads to a suppression of autophagy and autophagosomal degradation, which results in the persistence of damaged mitochondria in cells that in turn leads to a dysfunctional intracellular environment (Brownstein et al., 2017). These authors reported a decrease in the autophagy related gene transcripts, which is consistent with our results that show a hyper-methylation in the gene body of ATG16L2. This gene has also been shown to be differentially methylated in blood mononuclear cells of patients affected by multiple sclerosis compared with controls (Kulakova et al., 2016).

Calcium voltage-gated channel subunit alpha1 C (*CACNA1C)*, hyper-methylated in Angus during the heat stress period, belongs to a gene family coding for calcium channels. These channels transport positively charged calcium ions that play a key role in the cell’s ability to generate and transmit electrical signals (Liao et al., 2005). Calcium signaling is essential for T cell activation, tolerance of self-antigens, and homeostasis (Lewis, 2001). In lymphocytes, it has been shown that a signaling cascade leads to an increase of intracellular free calcium that in turn activate immune receptors (Fracchia et al., 2013). White blood cells of mice exposed to acute stress conditions, such as sleep deprivation, progressively loose intracellular Ca2 + and deplete endoplasmic reticulum (ER) reserves, resulting in an impaired immune response (Lungato et al., 2012). Genetic variations in CACNA1C are associated with multiple forms of neuropsychiatric diseases linked to stress and anxiety (Lee et al., 2016).

Growth arrest and DNA damage inducible alpha (*GADD45A)* was hyper-methylated in Angus during the challenge. Under various stress types, GADD45A maintains genomic integrity in many cell types by surveillance of a DNA-damage (Zhan, 2005) and promoting cell death, cell cycle arrest, and DNA repair (Moskalev et al., 2012). Increased mRNA levels of GADD45A have been reported in hematopoietic stem cells (HSCs), that are required for the continuous regeneration of the blood and the immune system and regulate stress responses (Orkin and Zon, 2008).

Changes in MAPK signaling, a pathway significantly enriched in DMGs in Angus during heat stress, are correlated with defects in innate immune responses of neutrophils and macrophages lacking GADD45 family members (Salerno et al., 2010).

## Conclusion

In summary, the present study describes DNA methylation profiles in blood samples of Nellore and Angus steers exposed to the sun during the high temperature season of the Brazilian summer. We detected regions that were differentially methylated that have been previously been associated with activation of immune responses and counteracting the effect of heat stress. Methylation analysis identified specific breed patterns with different methylation responses to stress and recovery. The Nellore appears to actively respond to high temperature by reducing methylation of several key genes and pathways associated with heat response. Angus, however, increases the methylation levels in genes in some of the few pathways that are common between breeds. Further research is also needed to explore the function of demethylation of non-CpG dinucleotides, to improve our knowledge about the biological significance of changes seen as a response to stress.

## Data Availability Statement

The datasets presented in this study can be found in online repositories. The names of the repository/repositories and accession number(s) can be found below: https://www.ncbi.nlm.nih.gov/geo/query/acc.cgi?acc=GSE161113, GSE161113.

## Ethics Statement

The animal study was reviewed and approved by CEUA (Ethics Committee on the Use of Animals) of the Universidade Estadual Paulista (FOA n° 2014-01445). Written informed consent was obtained from the owners for the participation of their animals in this study.

## Author Contributions

GN, JG, and PA-M conceived and designed the study. LZ, MM, AU, and GN performed the experiments. EC performed the DNA extraction, libraries preparation, and sequencing. MD and BL analyzed the data. MD, BL, YU, AS, GN, and PA-M prepared the manuscript. All authors reviewed and approved the final manuscript.

## Conflict of Interest

The authors declare that the research was conducted in the absence of any commercial or financial relationships that could be construed as a potential conflict of interest.

## Footnotes

1 http://www.nxt-dx.com/

2 http://www.bioinformatics.babraham.ac.uk/projects/fastqc/

3 http://www.bioinformatics.babraham.ac.uk/projects/trim_galore/

4 http://broadinstitute.github.io/picard

5 http://ftp.ensembl.org/pub/release-101/gff3/bos_taurus/Bos_taurus.ARS-UCD1.2.101.gff3.gz

## Supplementary Material

Supplementary Figure 1 DNA methylation levels of different functional regions in Angus and Nellore and in challenge and recovery periods.

Supplementary Figure 2 Principal component analysis (PCA) based on genome-wide DNA methylation in Angus and Nellore and in challenge (C) and recovery (R) periods.

Supplementary Table 1 Sequencing summary.

Supplementary Table 2 Differentially methylated regions and genes between challenge and recovery period in Angus and Nellore breeds (q value = 0.05). Sheet 1: shared regions between Angus and Nellore. Sheet 2: Angus unique regions. Sheet 3: Nellore unique regions.

Supplementary File 1 Details on animals treatment and diet.

## References

[B1] Aguilar I. Misztal I. Tsuruta S. (2010). Short communication: genetic trends of milk yield under heat stress for US Holsteins. *J. Dairy Sci*. 93 1754–1758. 10.3168/jds.2009-2756 20338455

[B2] Akalin A. Kormaksson M. Li S. Garrett-Bakelman F. E. Figueroa M. E. Melnick A. (2012). methylKit: a comprehensive R package for the analysis of genome-wide DNA methylation profiles. *Genome Biol*. 13:R87. 10.1186/gb-2012-13-10-r87 23034086 PMC3491415

[B3] Alemu T. W. Pandey H. O. Salilew Wondim D. Gebremedhn S. Neuhof C. Tholen E. (2018). Oxidative and endoplasmic reticulum stress defense mechanisms of bovine granulosa cells exposed to heat stress. *Theriogenology* 110 130–141. 10.1016/j.theriogenology.2017.12.042 29396041

[B4] Altan Ö Pabuccuoglu A. Altan A. Konyalioglu S. Bayraktar H. (2003). Effect of heat stress on oxidative stress, lipid peroxidation and some stress parameters in broilers. *Brit. Poult. Sci*. 44 545–550. 10.1080/00071660310001618334 14584844

[B5] Bagath M. Krishnanan G. Devaraj C. Rashamol V. P. Pragna P. Lees A. M. (2019). The impact of heat stress on the immune system in dairy cattle: a review. *Res. Vet. Sci.* 126 94–102. 10.1016/j.rvsc.2019.08.011 31445399

[B6] Bernabucci U. Biffani S. Buggiotti L. Vitali A. Lacetera N. Nardone A. (2014). The effects of heat stress in Italian Holstein dairy cattle. *J. Dairy Sci*. 97 471–486. 10.3168/jds.2013-6611 24210494

[B7] Bernstein E. Goldberg A. D. Allis C. D. (2007). Epigenetics: a landscape takes shape. *Cell* 128 635–638. 10.1016/j.cell.2007.02.006 17320500

[B8] Biffani S. Bernabucci U. Vitali A. Lacetera N. Nardone A. (2016). Short communication: effect of heat stress on nonreturn rate of Italian Holstein cows. *J. Dairy Sci*. 99 5837–5843. 10.3168/jds.2015-10491 27108174

[B9] Boneschansker L. Nakayama H. Eisenga M. Wedel J. Klagsbrun M. Irimia D. (2016). Netrin-1 augments chemokinesis in CD4+ T cells in vitro and elicits a proinflammatory response in vivo. *J. Immunol*. 197 1389–1398. 10.4049/jimmunol.1502432 27430720 PMC4976028

[B10] Braun P. R. Han S. Hing B. Nagahama Y. Gaul L. N. Heinzman J. T. (2019). Genome-wide DNA methylation comparison between live human brain and peripheral tissues within individuals. *Transl. Psychiatry* 9:47. 10.1038/s41398-019-0376-y PMC6355837 30705257

[B11] Brownstein A. J. Ganesan S. Summers C. M. Pearce S. Hale B. J. Ross J. W. (2017). Heat stress causes dysfunctional autophagy in oxidative skeletal muscle. *Physiol. Rep*. 5:e13317. 10.14814/phy2.13317 28646096 PMC5492206

[B12] Chovatiya R. Medzhitov R. (2014). Stress, inflammation, and defense of homeostasis. *Mol. Cell* 54 281–288. 10.1016/j.molcel.2014.03.030 24766892 PMC4048989

[B13] Collier R. J. Collier J. L. Rhoads R. P. Baumgard L. H. (2008). Invited review: genes involved in the bovine heat stress response. *J. Dairy Sci*. 91 445–454. 10.3168/jds.2007-0540 18218730

[B14] Cosma M. P. Tanaka T. Nasmyth K. (2010). Ordered recruitment of transcription and chromatin remodeling factors to a cell cycle- and developmentally regulated promoter. *Cell* 97 299–311. 10.1016/s0092-8674(00)80740-0 10319811

[B15] Cowley S. Whittaker K. Malone M. Donetto S. Grigulis A. Maben J. (2015). Why health visiting? Examining the potential public health benefits from health visiting practice within a universal service: a narrative review of the literature. *Int. J. Nurs. Stud.* 52 465–480. 10.1016/j.ijnurstu.2014.07.013 25304286

[B16] Cree I. A. Uttley L. Buckley Woods H. Kikuchi H. Reiman A. Harnan S. (2017). The evidence base for circulating tumour DNA blood-based biomarkers for the early detection of cancer: a systematic mapping review. *BMC Cancer* 17:697. 10.1186/s12885-017-3693-7 29061138 PMC5654013

[B17] Danaher R. J. Jacob R. J. Chorak M. D. Freeman C. S. Miller C. S. (1999). Heat stress activates production of herpes simplex virus type 1 from quiescently infected neurally differentiated PC12 cells. *J. Neurovirol.* 5 374–383. 10.3109/13550289909029478 10463859

[B18] Del Corvo M. Bongiorni S. Stefanon B. Sgorlon S. Valentini A. Ajmone Marsan P. (2020). Genome-wide DNA methylation and gene expression profiles in cows subjected to different stress level as assessed by cortisol in milk. *Genes* 11:850. 10.3390/genes11080850 32722461 PMC7464205

[B19] Dikmen S. Alava E. Pontes E. Fear J. M. Dikmen B. Y. Olson T. A. (2008). Differences in thermoregulatory ability between slick-haired and wild-type lactating Holstein cows in response to acute heat stress. *J. Dairy Sci.* 91 3395–3402. 10.3168/jds.2008-1072 18765598

[B20] Dikmen S. Cole J. B. Null D. J. Hansen P. J. (2012). Heritability of rectal temperature and genetic correlations with production and reproduction traits in dairy cattle. *J. Dairy Sci*. 95 3401–3405. 10.3168/jds.2011-4306 22612974

[B21] Dikmen S. Cole J. B. Null D. J. Hansen P. J. (2013). Genome-wide association mapping for identification of quantitative trait loci for rectal temperature during heat stress in Holstein cattle. *PLoS One* 8:e69202. 10.1371/journal.pone.0069202 23935954 PMC3720646

[B22] Dikmen S. Khan F. A. Huson H. J. Sonstegard T. S. Moss J. I. Dahl G. E. (2014). The SLICK hair locus derived from Senepol cattle confers thermotolerance to intensively managed lactating Holstein cows. *J. Dairy Sci.* 97 5508–5552. 10.3168/jds.2014-8087 24996281

[B23] Dikmen S. Wang X. Z. Ortega M. S. Cole J. B. Null D. J. Hansen P. J. (2015). Single nucleotide polymorphisms associated with thermoregulation in lactating dairy cows exposed to heat stress. *J. Anim. Breed. Genet.* 132 409–419. 10.1111/jbg.12176 26198991

[B24] Faylon M. P. Baumgard L. H. Rhoads R. P. Spurlock D. M. (2015). Effects of acute heat stress on lipid metabolism of bovine primary adipocytes. *J. Dairy Sci*. 98 8732–8740. 10.3168/jds.2015-9692 26433410

[B25] Flensner G. Ek A. C. Söderhamn O. Landtblom A. M. (2011). Sensitivity to heat in MS patients: a factor strongly influencing symptomology–an explorative survey. *BMC Neurol.* 11:27. 10.1186/1471-2377-11-27 21352533 PMC3056752

[B26] Fracchia K. M. Pai C. Y. Walsh C. M. (2013). Modulation of T cell metabolism and function through calcium signaling. *Front. Immunol.* 4:324. 10.3389/fimmu.2013.00324 24133495 PMC3795426

[B27] Georges M. Charlier C. Cockett N. (2003). The callipyge locus: evidence for the trans interaction of reciprocally imprinted genes. *Trends Genet.* 19 248–252. 10.1016/s0168-9525(03)00082-9 12711215

[B28] Gholami H. Chamani M. Towhidi A. Fazeli M. H. (2011). Improvement of semen quality in Holstein bulls during heat stress by dietary supplementation of omega-3 fatty acids. *Int. J. Fertil. Steril*. 4 160–167. 24851176 PMC4023502

[B29] Gomides L. F. Duarte I. D. Ferreira R. G. Perez A. C. Francischi J. N. Klein A. (2012). Proteinase-activated receptor-4 plays a major role in the recruitment of neutrophils induced by trypsin or carrageenan during pleurisy in mice. *Pharmacology* 89 275–282. 10.1159/000337378 22517275

[B30] Hämäläinen P. Ikonen A. Romberg A. Helenius H. Ruutiainen J. (2012). The effects of heat stress on cognition in persons with multiple sclerosis. *Mult. Scler.* 18 489–497. 10.1177/1352458511422926 21914688

[B31] Hayes B. J. Bowman P. J. Chamberlain A. J. Savin K. van Tassell C. P. Sonstegard T. S. (2009). A validated genome wide association study to breed cattle adapted to an environment altered by climate change. *PLoS One* 4:e6676. 10.1371/journal.pone.0006676 19688089 PMC2722733

[B32] Henriksen T. V. Reinert T. Christensen E. Sethi H. Birkenkamp-Demtröder K. Gögenur M. (2020). The effect of surgical trauma on circulating free DNA levels in cancer patients-implications for studies of circulating tumor DNA. *Mol. Oncol.* 14 1670–1679. 10.1002/1878-0261.12729 32471011 PMC7400779

[B33] Holbro T. Hynes N. E. (2004). ErbB receptors: directing key signaling networks throughout life. *Annu. Rev. Pharmacol. Toxicol.* 44 195–217. 10.1146/annurev.pharmtox.44.101802.121440 14744244

[B34] Ishibashi K. Fujita N. Kanno E. Omori H. Yoshimori T. Itoh T. (2011). Atg16L2, a novel isoform of mammalian Atg16L that is not essential for canonical autophagy despite forming an Atg12–5-16L2 complex. *Autophagy* 7 1500–1513. 10.4161/auto.7.12.18025 22082872 PMC3288023

[B35] Kataoka H. Hamilton J. R. McKemy D. D. Camerer E. Zheng Y. W. Cheng A. (2003). Protease-activated receptors 1 and 4 mediate thrombin signaling in endothelial cells. *Blood* 102 3224–3231. 10.1182/blood-2003-04-1130 12869501

[B36] Knoops B. Argyropoulou V. Becker S. Ferté L. Kuznetsova O. (2016). Multiple roles of peroxiredoxins in inflammation. *Mol. Cells* 39 60–64. 10.14348/molcells.2016.2341 26813661 PMC4749876

[B37] Kronenberg M. Gapin L. (2002). The unconventional lifestyle of NKT cells. *Nat. Rev. Immunol.* 2 557–568. 10.1038/nri854 12154375

[B38] Kronenberg M. Gapin L. (2007). Natural killer T cells: know thyself. *Proc. Natl. Acad. Sci. U.S.A.* 104 5713–5714. 10.1073/pnas.0701493104 17389403 PMC1851556

[B39] Krueger F. Andrews S. R. (2011). Bismark: a flexible aligner and methylation caller for Bisulfite-Seq applications. *Bioinformatics* 27 1571–1572. 10.1093/bioinformatics/btr167 21493656 PMC3102221

[B40] Kulakova O. G. Kabilov M. R. Danilova L. V. Popova E. V. Baturina O. A. Tsareva E. Y. (2016). Whole-genome DNA methylation analysis of peripheral blood mononuclear cells in multiple sclerosis patients with different disease courses. *Acta Naturae* 8 103–110. 10.32607/20758251-2016-8-3-103-110 PMC5081712 27795849

[B41] Langmead B. Salzberg S. L. (2012). Fast gapped-read alignment with Bowtie 2. *Nat. Methods* 9 357–359. 10.1038/nmeth.1923 22388286 PMC3322381

[B42] Lee A. S. De Jesús-Cortés H. Kabir Z. D. Knobbe W. Orr M. Burgdorf C. (2016). The Neuropsychiatric disease-associated gene cacna1c mediates survival of young hippocampal neurons. *eNeuro* 3:ENEURO.0006-16.2016. 10.1523/ENEURO.0006-16.2016 27066530 PMC4819284

[B43] Leger A. J. Covic L. Kuliopulos A. (2006). Protease-activated receptors in cardiovascular diseases. *Circulation* 114 1070–1077. 10.1161/circulationaha.105.574830 16952995

[B44] Lewis R. S. (2001). Calcium signaling mechanisms in T lymphocytes. *Annu. Rev. Immunol*. 19 497–521. 11244045 10.1146/annurev.immunol.19.1.497

[B45] Li M. Wu H. Luo Z. Xia Y. Guan J. Wang T. (2012). An atlas of DNA methylomes in porcine adipose and muscle tissues. *Nat. Commun.* 3:850. 10.1038/ncomms1854 22617290 PMC3508711

[B46] Liao P. Yong T. F. Liang M. C. Yue D. T. Soong T. W. (2005). Splicing for alternative structures of Cav1.2 Ca2+ channels in cardiac and smooth muscles. *Cardiovasc. Res*. 68 197–203. Epub. 2005 Jul 27. Review., 10.1016/j.cardiores.2005.06.024 16051206

[B47] Lin H. Decuypere E. Buyse J. (2006). Acute heat stress induces oxidative stress in broiler chickens. *Comp. Biochem. Physiol. A Mol. Integr. Physiol*. 144 11–17. 10.1016/j.cbpa.2006.01.032 16517194

[B48] Littlejohn M. D. Henty K. M. Tiplady K. Johnson T. Harland C. Lopdell T. (2014). Functionally reciprocal mutations of the prolactin signalling pathway define hairy and slick cattle. *Nat. Commun.* 5:5861. 10.1038/ncomms6861 25519203 PMC4284646

[B49] Lungato L. Gazarini M. L. Paredes-Gamero E. J. Tersariol I. L. Tufik S. D’Almeida V. (2012). Sleep deprivation impairs calcium signaling in mouse splenocytes and leads to a decreased immune response. *Biochim. Biophys. Acta* 1820 1997–2006. 10.1016/j.bbagen.2012.09.010 23000491

[B50] Ly N. P. Komatsuzaki K. Fraser I. P. Tseng A. A. Prodhan P. Moore K. J. (2005). Netrin-1 inhibits leukocyte migration in vitro and in vivo. *Proc. Natl. Acad. Sci. U.S.A.* 102 14729–14734. 10.1073/pnas.0506233102 16203981 PMC1253572

[B51] Ma B. Wilker E. H. Willis-Owen S. A. Byun H. M. Wong K. C. Motta V. (2014). Predicting DNA methylation level across human tissues. *Nucleic Acids Res.* 42 3515–3528. 10.1093/nar/gkt1380 24445802 PMC3973306

[B52] Macciotta N. P. P. Biffani S. Bernabucci U. Lacetera N. Vitali A. Ajmone-Marsan P. (2017). Derivation and genome-wide association study of a principal component-based measure of heat tolerance in dairy cattle. *J. Dairy Sci*. 100 4683–4697. 10.3168/jds.2016-12249 28365122

[B53] McDowell R. E. Hooven N. W. Camoens J. K. (1976). Effect of climate on performance of Holsteins in first lactation. *J. Dairy Sci.* 59 965–973. 10.3168/jds.S0022-0302(76)84305-6

[B54] Mehla K. Magotra A. Choudhary J. Singh A. K. Mohanty A. K. Upadhyay R. C. (2014). Genome-wide analysis of the heat stress response in Zebu (Sahiwal) cattle. *Gene* 533 500–507. 10.1016/j.gene.2013.09.051 24080481

[B55] Mitchell G. Amit I. Garber M. French C. Lin M. F. Feldser D. (2009). Chromatin signature reveals over a thousand highly conserved large non-coding RNAs in mammals. *Nature* 458 223–227. 10.1038/nature07672 19182780 PMC2754849

[B56] Moskalev A. A. Smit-McBride Z. Shaposhnikov M. V. Plyusnina E. N. Zhavoronkov A. Budovsky A. (2012). Gadd45 proteins: relevance to aging, longevity and age-related pathologies. *Ageing Res. Rev*. 11 51–66. 10.1016/j.arr.2011.09.003 21986581 PMC3765067

[B57] Mujahid A. Yoshiki Y. Akiba Y. Toyomizu M. (2005). Superoxide radical production in chicken skeletal muscle induced by acute heat stress. *Poult. Sci*. 84 307–314. 10.1093/ps/84.2.307 15742968

[B58] Nagai M. Iriki M. (2001). “Changes in immune activities by heat stress,” in *Thermotherapy for Neoplasia, Inflammation, and Pain*, eds Kosaka M. Sugahara T. Schmidt K. L. Simon E. (Tokyo: Springer), 266–270 10.1007/978-4-431-67035-3_30

[B59] Nardone A. Ronchi B. Lacetera N. Ranieri M. S. Bernabucci U. (2010). Effects of climate changes on animal production and sustainability of livestock systems. *Livest. Sci.* 130 57–69. 10.1016/j.livsci.2010.02.011

[B60] Nassour H. Wang Z. Saad A. Papaluca A. Brosseau N. Affar el B. (2016). Peroxiredoxin 1 interacts with and blocks the redox factor APE1 from activating interleukin-8 expression. *Sci. Rep.* 6:29389. 10.1038/srep29389 27388124 PMC4937415

[B61] Nätt D. Rubin C.-J. Wright D. Johnsson M. Beltéky J. Andersson L. (2012). Heritable genome-wide variation of gene expression and promoter methylation between wild and domesticated chickens. *BMC Genomics* 13:59. 10.1186/1471-2164-13-59 22305654 PMC3297523

[B62] Olson T. A. Lucena C. Chase C. C. Jr. Hammond A. C. (2003). Evidence of a major gene influencing hair length and heat tolerance in *Bos taurus* cattle. *J. Anim. Sci.* 81 80–90. 10.2527/2003.81180x 12597376

[B63] Orkin S. H. Zon L. I. (2008). Hematopoiesis: an evolving paradigm for stem cell biology. *Cell* 132 631–644. 10.1016/j.cell.2008.01.025 18295580 PMC2628169

[B64] Pelidou S. H. Tsifetaki N. Giannopoulos S. Deretzi G. Voulgari P. Kyritsis A. (2007). Multiple sclerosis associated with systemic sclerosis. *Rheumatol. Int*. 27 771–773. 17171345 10.1007/s00296-006-0282-5

[B65] Perano K. M. Usack J. G. Angenent L. T. Gebremedhin K. G. (2015). Production and physiological responses of heat-stressed lactating dairy cattle to conductive cooling. *J. Dairy Sci.* 98 5252–5261. 10.3168/jds.2014-8784 26074243

[B66] Pham T. T. Angus S. P. Johnson G. L. (2013). MAP3K1: genomic alterations in cancer and function in promoting cell survival or apoptosis. *Genes Cancer* 4 419–426. 10.1177/1947601913513950 24386504 PMC3877667

[B67] Porto-Neto L. R. Bickhart D. M. Landaeta-Hernandez A. J. Utsunomiya Y. T. Pagan M. Jimenez E. (2018). Convergent evolution of Slick coat in cattle through truncation mutations in the prolactin receptor. *Front. Genet.* 9:57. 10.3389/fgene.2018.00057 29527221 PMC5829098

[B68] Rajaud A. Noblet-Ducoudré N. (2017). Tropical semi-arid regions expanding over temperate latitudes under climate change. *Clim. Change* 144 703–719. 10.1007/s10584-017-2052-7

[B69] Rhoads M. L. Kim J. W. Collier R. J. Crooker B. A. Boisclair Y. R. Baumgard L. H. (2010). Effects of heat stress and nutrition on lactating Holstein cows: II. *J. Dairy Sci.* 93 170–179. 10.3168/jds.2009-2469 20059916

[B70] Rojas-Downing M. M. Nejadhashemi P. A. Timothy H. Woznicki S. A. (2017). Climate change and livestock: impacts, adaptation, and mitigation. *Clim. Risk. Manag.* 16 145–163.

[B71] Salerno D. Tront S. J. Hoffman B. Liebermann D. (2010). Deficiency in stress response Gadd45a and Gadd45b alters MAPK kinase signaling leading to functional defects in innate immune responses of neutrophils and macrophages in vitro and in vivo. *Blood* 116:927. 10.1182/blood.v116.21.927.927

[B72] Scheiermann C. Kunisaki Y. Frenette P. S. (2013). Circadian control of the immune system. *Nat. Rev. Immunol*. 13 190–198. 10.1038/nri3386 23391992 PMC4090048

[B73] Sibbons C. M. Irvine N. A. Pérez-Mojica J. E. Calder P. C. Lillycrop K. A. Fielding B. A. (2018). Polyunsaturated fatty acid biosynthesis involving Δ8 desaturation and differential dna methylation of *FADS2* regulates proliferation of human peripheral blood mononuclear cells. *Front. Immunol.* 9:432. 10.3389/fimmu.2018.00432 29556240 PMC5844933

[B74] Singh K. Erdman R. A. Swanson K. M. Molenaar A. J. Maqbool N. J. Wheeler T. T. (2010). Epigenetic regulation of milk production in dairy cows. *J. Mammary Gland Biol. Neoplasia* 15 101–112. 10.1007/s10911-010-9164-2 20131087

[B75] Skibiel A. L. Peñagaricano F. Amorín R. Ahmed B. M. Dahl G. E. Laporta J. (2018). In utero heat stress alters the offspring epigenome. *Sci. Rep.* 8:14609. 10.1038/s41598-018-32975-1 30279561 PMC6168509

[B76] Steinhoff M. Buddenkotte J. Shpacovitch V. Rattenholl A. Moormann C. Vergnolle N. (2005). Proteinase-activated receptors: transducers of proteinase-mediated signaling in inflammation and immune response. *Endocr. Rev*. 26 1–43. 10.1210/er.2003-0025 15689571

[B77] Strahl B. D. Allis C. D. (2000). The language of covalent histone modifications. *Nature* 403 41–45. 10.1038/47412 10638745

[B78] Suddason T. Anwar S. Charlaftis N. Gallagher E. T. - (2016). Cell-specific deletion of Map3k1 reveals the critical role for Mekk1 and Jnks in Cdkn1b-dependent proliferative expansion. *Cell Rep*. 14 449–457. 10.1016/j.celrep.2015.12.047 26774476 PMC4733086

[B79] Thom E. C. (1959). The discomfort index. *Weatherwise* 12 57–59. 10.1080/00431672.1959.9926960

[B80] Valente T. S. Sant’Anna A. C. Baldi F. Albuquerque L. G. da Costa M. J. (2015). Genetic association between temperament and sexual precocity indicator traits in Nellore cattle. *J. Appl. Genet.* 56 349–354. 10.1007/s13353-014-0259-0 25472773

[B81] Van Laere A. S. Nguyen M. Braunschweig M. Nezer C. Collette C. Moreau L. (2003). A regulatory mutation in IGF2 causes a major QTL effect on muscle growth in the pig. *Nature* 425 832–836. 10.1038/nature02064 14574411

[B82] Vergnolle N. Derian C. K. D’Andrea M. R. Steinhoff M. Andrade-Gordon P. (2002). Characterization of thrombin-induced leukocyte rolling and adherence: a potential proinflammatory role for proteinase-activated receptor-4. *J. Immunol*. 169 1467–1473. 10.4049/jimmunol.169.3.1467 12133973

[B83] von Boehmer H. (1990). Developmental biology of T cells in T cell-receptor transgenic mice. *Annu. Rev. Immunol*. 8 531–556. 10.1146/annurev.iy.08.040190.002531 2188673

[B84] Vuocolo T. Byrne K. White J. McWilliam S. Reverter A. Cockett N. E. (2007). Identification of a gene network contributing to hypertrophy in callipyge skeletal muscle. *Physiol. Genomics* 28 253–272. 10.1152/physiolgenomics.00121.2006 17077277

[B85] Wang H. Tuominen L. K. Tsai C. (2011). SLIM: a sliding linear model for estimating the proportion of true null hypotheses in datasets with dependence structures. *Bioinformatics* 27 225–231. 10.1093/bioinformatics/btq650 21098430

[B86] Wehbi V. L. Taskén K. (2016). Molecular mechanisms for cAMP-mediated immunoregulation in T cells–role of anchored protein kinase a signaling units. *Front. Immunol*. 7:222. 10.3389/fimmu.2016.00222 27375620 PMC4896925

[B87] Weyrich A. Benz S. Karl S. Jeschek M. Jewgenow K. Fickel J. (2016). Paternal heat exposure causes DNA methylation and gene expression changes of Stat3 in Wild guinea pig sons. *Ecol. Evol*. 6 2657–2666. 10.1002/ece3.1993 27066228 PMC4769883

[B88] Wu B. Zhang S. Guo Z. Wang G. Zhang G. Xie L. (2018). RAS P21 protein activator 3 (RASA3) specifically promotes pathogenic T helper 17 cell generation by repressing T-helper-2-cell-biased programs. *Immunity* 49 886–898.e5. 10.1016/j.immuni.2018.09.004 30446383 PMC6249088

[B89] Xu W. F. Andersen H. Whitmore T. E. Presnell S. R. Yee D. P. Ching A. (1998). Cloning and characterization of human protease-activated receptor 4. *Proc. Natl. Acad. Sci. U.S.A.* 95 6642–6646. 9618465 10.1073/pnas.95.12.6642 PMC22580

[B90] Yoshihara T. Naito H. Kakigi R. Ichinoseki-Sekine N. Ogura Y. Sugiura T. (2013). Heat stress activates the Akt/mTOR signalling pathway in rat skeletal muscle. *Acta Physiol.* 207 416–426. 10.1111/apha.12040 23167446

[B91] Yu G. Wang L. Han Y. He Q. (2012). clusterProfiler: an R package for comparing biological themes among gene clusters. *OMICS* 16 284–287. 10.1089/omi.2011.0118 22455463 PMC3339379

[B92] Zaccolo M. Movsesian M. A. (2007). cAMP and cGMP signaling cross-talk. *Circ. Res*. 100 1569–1578. 10.1161/circresaha.106.144501 17556670

[B93] Zhan Q. (2005). Gadd45a, a p53- and BRCA1-regulated stress protein, in cellular response to DNA damage. *Mutat. Res*. 569 133–143. 10.1016/j.mrfmmm.2004.06.055 15603758

[B94] Zhang J. Song F. Zhao X. Jiang H. Wu X. Wang B. (2017). EGFR modulates monounsaturated fatty acid synthesis through phosphorylation of SCD1 in lung cancer. *Mol. Cancer* 16:127. 10.1186/s12943-017-0704-x 28724430 PMC5518108

[B95] Zhu H. Zhu C. Mi W. Chen T. Zhao H. Zuo X. (2018). Integration of genome-wide DNA methylation and transcription uncovered aberrant methylation-regulated genes and pathways in the peripheral blood mononuclear cells of systemic sclerosis. *Int. J. Rheumatol.* 2018:7342472. 10.1155/2018/7342472 30245726 PMC6139224

