# Supplementary figures and images for: Methylome Patterns of Cattle Adaptation to Heat Stress
Source: Front Genet. 2021 May 28;12:633132. doi: 10.3389/fgene.2021.633132 (PMC8194315; doi:10.3389/fgene.2021.633132)

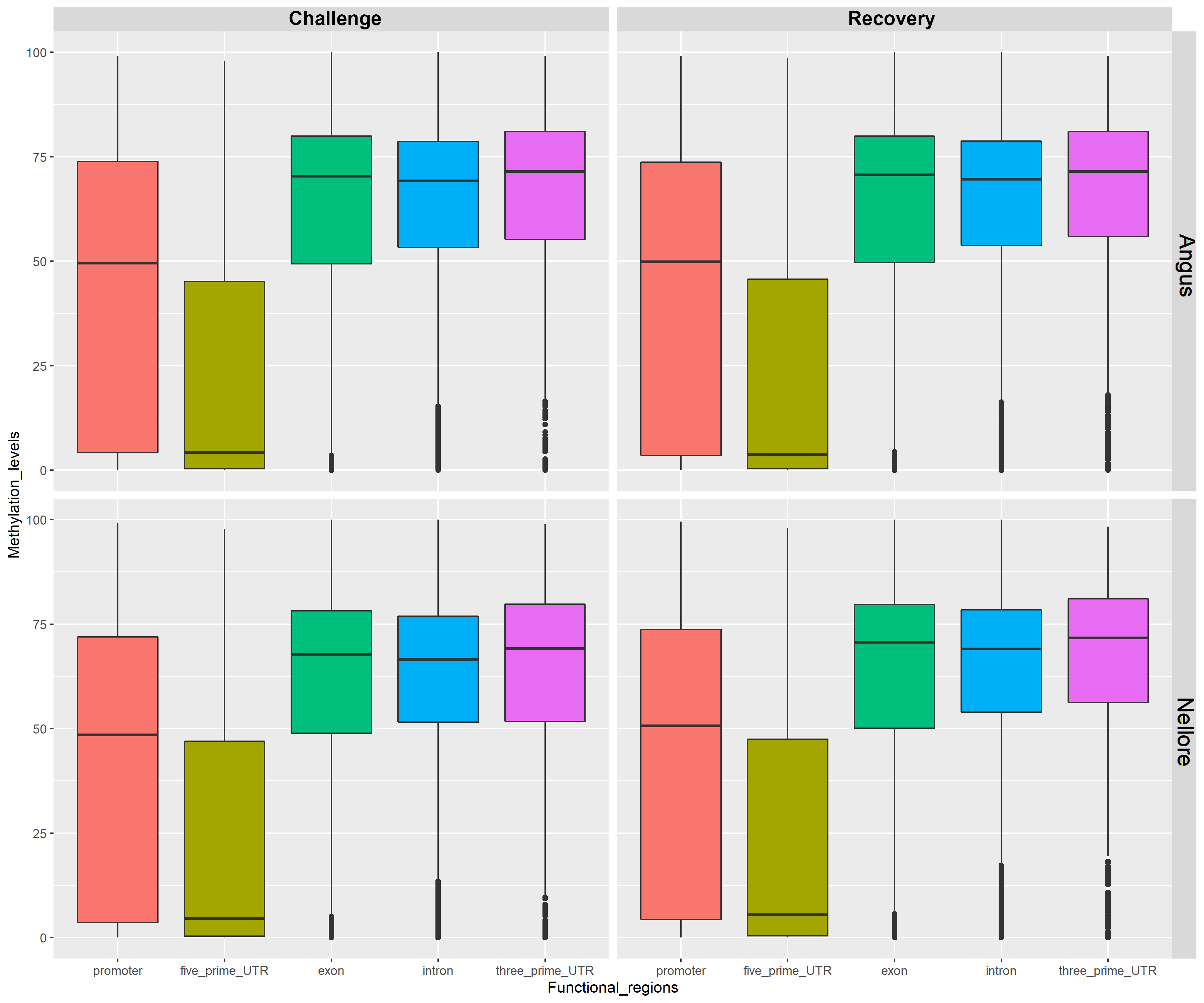

Supplement: Supplementary Figure 1 — DNA methylation levels of different functional regions in Angus and Nellore and in challenge and recovery periods. [file Image_1.TIFF]

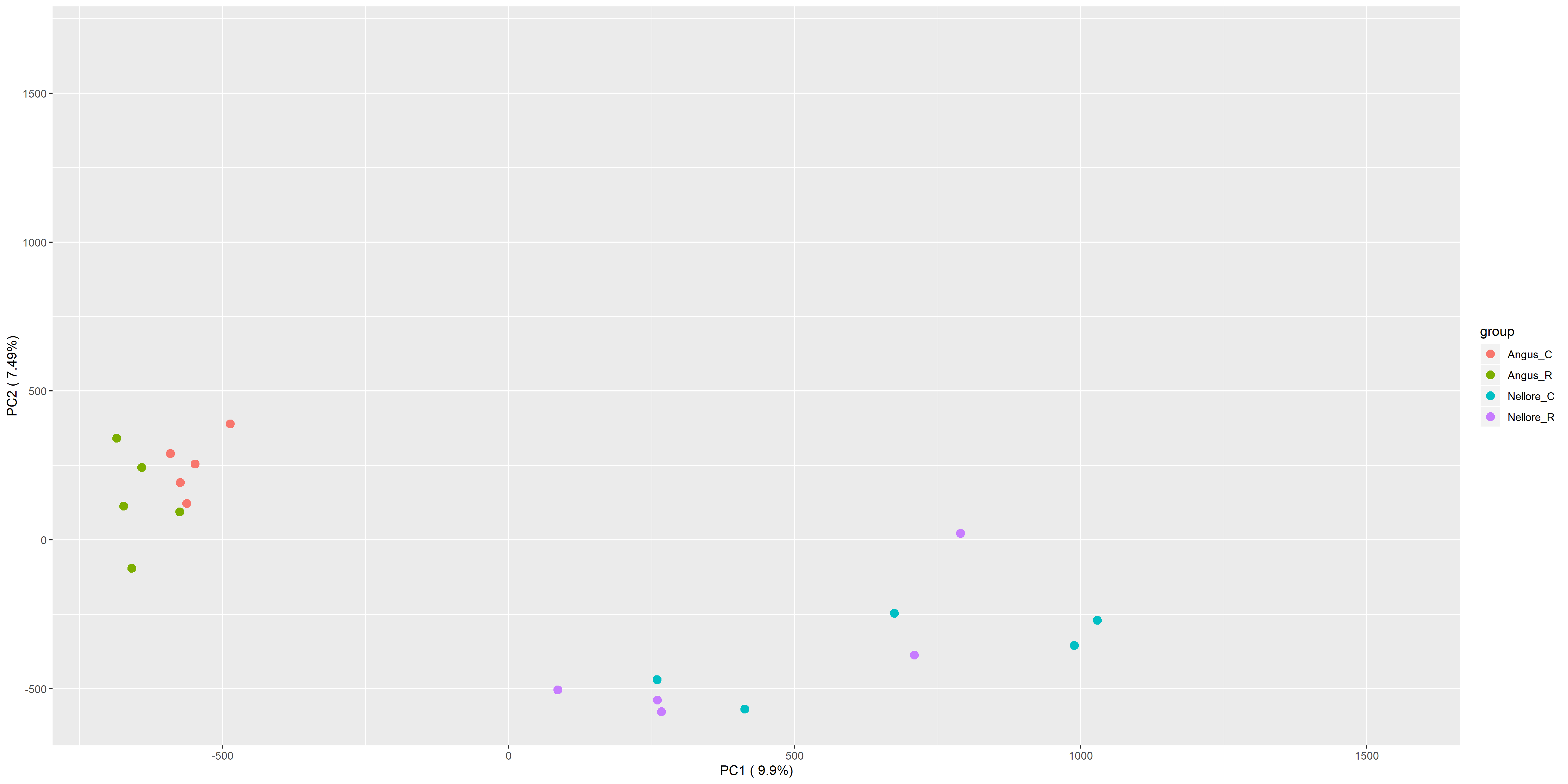

Supplement: Supplementary Figure 2 — Principal component analysis (PCA) based on genome-wide DNA methylation in Angus and Nellore and in challenge (C) and recovery (R) periods. [file Image_2.TIFF]
