# Supplementary material for: Methylome Patterns of Cattle Adaptation to Heat Stress
Source: Front Genet. 2021 May 28;12:633132. doi: 10.3389/fgene.2021.633132 (PMC8194315; doi:10.3389/fgene.2021.633132)
Supplement: Supplementary File 1 — Details on animals treatment and diet. [file Data_Sheet_1.pdf]

Supplementary file 1. Animals treatment and diet details

Chemoprophylactic measures were applied for the prevention of erythemia and babesiosis, diseases whose vector Rhipicephalus microplus has wide dissemination in the region.

Therefore, as soon as they arrived from Rio Grande do Sul, Angus were administered a treatment consisting of 7% diminazenedi aceturate (1ml / 60kg) and oxytetracycline 20% (1ml / 30kg), in addition to being vaccinated for Infectious Bovine Rhinotracheitis (IBR), Bovine Viral Diarrhea (BVDV), Leptospirosis and Clostridiosis. In a second moment, a chemoprophylactic treatment with repetitions was adopted every 45 days that consisted in 7% diminazenedi aceturate (1ml / 60kg), oxytetracycline 20% (1ml / 30kg), doramectin (1ml / 50kg) and fluazuron 2.5%.

In November all animals were vaccinated against foot-and-mouth disease, according to the Brazilian national vaccination calendar.

- ***Adaptation period***

From arrival until October 2, 2015 (>30 days since their arrival), animals were reared on *Brachiaria brizantha* pasture, with shadow available and water access. Gradually they were adapted to semi-confined and to a diet based on citrus pulp, ground corn, soybean meal and urea, as well as mineral supplementation, in the proportion of 2.5% of live weight. Finally they were confined to four lots with access to shade and received a same diet based on sugarcane bagasse 45 (Êxito Rural®, with a ratio forage: concentrate 55:45) administered twice a day and water ad libitum. On October 3 2015 they were divided in 4 homogeneous groups (2 Nellore and 2 Angus groups) of 12 or 13 animals and confined into four 200 square meter paddocks of which 100 square meters covered by a (80% sunblock) shadowing net until December 3rd(60 days).

- ***Challenge***

The experiment started on December 4th by removing the shadowing net from one of the Nellore and one of the Angus paddocks. Animal groups were therefore kept without shadow (Nellore-sun and Angus-sun groups) or with shadow available (Nellore-shadow and Angus-shadow groups) until February 3rd 2016 (56 days). Due to critical conditions of some animals,all were allowed to pasture during the night from December 25th 2015 to January 10th 2016.

- ***Recovery period***

On February 4th shadowing nets were re-put in place in all paddocks and animalswere kept with shadow available until slaughtering.
